# Cdk2 Silencing via a DNA/PCL Electrospun Scaffold Suppresses Proliferation and Increases Death of Breast Cancer Cells

**DOI:** 10.1371/journal.pone.0052356

**Published:** 2012-12-20

**Authors:** Clément Achille, Sowmya Sundaresh, Benjamin Chu, Michael Hadjiargyrou

**Affiliations:** 1 Institut Supérieur des Biosciences de Paris, Université de Paris Est Créteil, Créteil, France; 2 Department of Biomedical Engineering, Stony Brook University, Stony Brook, New York, United States of America; 3 Department of Chemistry, Stony Brook University, Stony Brook, New York, United States of America; 4 Department of Life Sciences, New York Institute of Technology, Old Westbury, New York, United States of America; Wayne State University, United States of America

## Abstract

RNA interference (RNAi) is a promising approach for cancer treatment. Site specific and controlled delivery of RNAi could be beneficial to the patient, while at the same time reducing undesirable off-target side effects. We utilized electrospinning to generate a biodegradable scaffold capable of incorporating and delivering a bioactive plasmid encoding for short hairpin (sh) RNA against the cell cycle specific protein, Cdk2. Three electrospun scaffolds were constructed, one using polycaprolactone (PCL) alone (Control) and PCL with plasmid DNA encoding for either Cdk2 (Cdk2i) and EGFP (EGFPi, also served as a control) shRNA. Scaffold fiber diameters ranged from 1 to 20 µm (DNA containing) and 0.2–3 µm (Control). While the electrospun fibers remained intact for more than two weeks in physiological buffer, degradation was visible during the third week of incubation. Approximately 20–60 ng/ml (∼2.5% cumulative release) of intact and bioactive plasmid DNA was released over 21 days. Further, Cdk2 mRNA expression in cells plated on the Cdk2i scaffold was decreased by ∼51% and 30%, in comparison with that of cells plated on Control or EGFPi scaffold, respectively. This decrease in Cdk2 mRNA by the Cdk2i scaffold translated to a ∼40% decrease in the proliferation of the breast cancer cell line, MCF-7, as well as the presence of increased number of dead cells. Taken together, these results represent the first successful demonstration of the delivery of bioactive RNAi-based plasmid DNA from an electrospun polymer scaffold, specifically, in disrupting cell cycle regulation and suppressing proliferation of cancer cells.

## Introduction

Conventional treatments for cancer using chemotherapy have serious side-effects because they are usually administered systemically and affect both normal and healthy tissues as well as cancer cells. To overcome this problem, RNAi is considered to be one of the most promising approaches because of its versatility [1]. Through several strategies that target cancer cells, specifically by down-regulating cell cycle required proteins (i.e. Cyclins, Cdks), suppressing oncogene expression, or inducing apoptosis, it is hoped that RNAi will be a successful and novel anti-cancer therapeutic. Despite these approaches, site-specific delivery of RNAi remains a major problem as the short interfering (si), short hairpin (sh) and microRNAs must remain bioactive and be able to enter the target cells. To this end, many laboratories are designing novel nonviral RNAi delivery approaches that range from use of liposomes [2], nanoparticles [3], gels [4] and scaffolds [5].

In the last decade, we have witnessed an explosion in the use of electrospun scaffolds for a myriad of applications, especially in tissue engineering and regenerative medicine [6], [7], as well as DNA and drug delivery systems [8], [9]. These scaffolds offer a number of advantages that include a high surface area to volume ratio with interconnected pores that mimic the topology of the extracellular matrix (ECM) and can be functionalized with biomolecules (which are often better protected from degradation). With the increase in porosity, the controlled variation in the degradation rate, and the versatility offered in the production process [9], [10], scaffolds can be fabricated with desirable characteristics enabling robust oxygen, nutrient, and waste transport through the scaffold, while at the same time permitting cell adhesion, migration, proliferation and differentiation. As such, the scaffolds are ideal constructs for tissue engineering and regenerative medicine applications [6], [7], as well as DNA and drug delivery systems [8], [9]. Collectively, these properties also enable the scaffolds to specifically serve their intended design and application [10].

The utility of electrospun scaffolds as drug and gene delivery systems was previously demonstrated by our laboratory using antibiotics [11], plasmid DNA [12]–[14] and proteins [15], [16]. Variations of these scaffolds as drug and gene delivery vehicles have also been subsequently reported by others [17]–[25]. More recently, and directly related to this study, two studies reported on the incorporation and release of siRNA oligonucleotides from electrospun PCL based scaffolds and their successful silencing of the housekeeping gene, GAPDH [26], [27]. Further, we extend our approach to test the feasibility of incorporating plasmid DNA encoding shRNA in an electrospun scaffold. Herein, we show that this plasmid DNA-based scaffold is capable of delivering bioactive DNA encoding for shRNA leading to the successful silencing of its target gene (Cdk2) and resulting in the disruption of the cell cycle, as well as a reduction in the proliferation and viability of MCF-7 human breast cancer cells. The data presented in this manuscript verify our approach and initial design of the electrospun gene delivery system and its success in delivering bioactive plasmid DNA encoding for shRNA as a possible means of suppressing cancer cell proliferation and inducing cell death. Lastly, our results represent the first successful demonstration of the delivery of bioactive RNAi-based plasmid from a polymeric electrospun scaffold.

## Materials and Methods

### Reagents

Plasmids, pKD-Cdk2-v5 (3,099 bp) and pKD-EGFP-v1 (3,099 bp), encoding for Cdk2 and EGFP short hairpin (sh) RNA, were purchased from Millipore (Lake Placid, NY). PicoGreen DNA reagent was purchased from Molecular Probes (Eugene, OR, USA). Polycaprolactone (PCL), tetrahydrofuran (THF) and N,N-dimethylformamide (DMF) were obtained from Sigma-Aldrich (St. Louis MO).

### Scaffold Fabrication

Purified plasmid DNA (∼800 µg) in TE buffer was added to a mixture of 50/50 DMF/THF (total of 10.75 ml) and was stirred for 8–24 hrs. Then, PCL (0.8 g per amount of 2.154 g of DMF and 2.154 g of THF) was added to the solution and the mixture was further stirred for an additional 24 hrs. The DNA/polymer solution was then transferred in a 20 ml plastic syringe fitted with a 20 gauge needle and set up in the electrospinning apparatus. Scaffolds were electrospun at 25 kV with a solution flow rate of 20 µl/min that was controlled via a programmable syringe pump (KD Scientific). As ∼85% of the DNA/polymer solution was used, then a total of ∼680 µg plasmid DNA was incorporated throughout each scaffold, and if we assume a uniform distribution of the plasmid DNA, then the total amount of plasmid DNA in each scaffold is ∼2.125 µg/cm^2^. Further, a rotating drum was used as collector (cathode) and was placed at ∼22 cm under the spinneret (anode), where the scaffolds were formed. Three different types of scaffolds were electrospun: 1) no DNA (Control); 2) one with pKD-Cdk2-v5 plasmid (Cdk2i); and 3) another with pKD-EGFP-v1 plasmid (EGFPi, also served as a control), and each scaffold measured ∼32×10 cm.

### Scaffold Characterization

Electrospun scaffold morphology was evaluated via Scanning Electron Microscopy (SEM). Images were obtained using a desktop Scanning Electron Microscope (Phenom, Eindhoven, Netherlands).

### Degradation Study

Two 1.0×1.0 cm pieces of each type of scaffold, one containing the experimental pKD-Cdk2-v5 plasmid (Cdk2i) and the other with no DNA (Control), were placed in 1 mL of 1X PBS buffer in 1.5 mL eppendorf tubes and incubated at 37°C under constant rotation (20 rpm). At each time point (days 8, 14 and 21 days), samples were removed from the solution, and a small piece (∼1 mm×2 mm) was cut, vacuum dried and stored at −20°C. The remaining scaffold pieces were put in a new tube containing the same amount of PBS and were further incubated. The same procedure was repeated at each time point tested. Lastly, all samples were imaged using SEM.

### DNA Release Assay

Nine 1.0×1.0 cm scaffold pieces and sets of three were added into 1 mL of 1X PBS buffer in 1.5 mL Eppendorf tubes. To ensure consistency between scaffolds, the 1.0×1.0 cm pieces were taken from the same random locations of the original Cdk2i and EFGPi (∼32×10 cm) scaffolds. The tubes containing the scaffolds were then incubated at 37°C under constant rotation (20 rpm). At each time point (days 1, 7, 14, and 21), the three scaffold pieces were removed from the solution and placed in a new tube containing the same amount of PBS. Collected samples were stored at −20°C until the end of the release study. The amount of released plasmid DNA from each set of scaffolds was quantified by using a PicoGreen assay. DNA concentration from each set of scaffolds was measured at 530 nm in a microplate reader (CytoFluor Series 4000, Perseptive Biosystems) and the values from the three sets were averaged. Finally, the measured amounts were compared to the predicted total quantity of DNA contained in each piece based on the theoretical calculation described in the Scaffold fabrication section.

### Gel Electrophoresis

Electrospun scaffolds containing pKD-Cdk2-v5 (Cdk2i) and pKD-EGF-v1 (EGFPi) plasmid DNA were cut into five 1.0×1.0 cm pieces, placed in 1 mL of 1X PBS buffer in 1.5 mL Eppendorf tubes and incubated at 37°C under shaking (20 rpm) for 21 days. The released DNA was then precipitated with 95% ethanol, washed with 70% ethanol, air dried and then resuspended in 10 µl of TE buffer. The entire solution was loaded on a 1% agarose gel containing ethidium bromide. For the unincorporated plasmids, 500 ng of DNA was used. The DNA was then visualized under UV light.

### Cell Culture and Transfection

Human breast cancer MCF-7 cells were maintained in 1X RPMI medium supplemented with 10% of FBS and 1% of 1X Streptomycin/Penicillin (Life Technologies, Grand Island, USA). Cells were plated into a six well plate at an initial density of 1×10^5^ cells per well 24 hours before transfection. Two wells received pKD-Cdk2-v5 plasmid DNA (mixed with Fugene6 transfection reagent, 3∶2 ratio of reagent (ml) to DNA (mg), another two received the same except using the pKD-EGFP-v1 plasmid and the last two did not receive any DNA and thus served as negative controls. The total amount of plasmid DNA used was 1 µg for each type/well. Cells were incubated at 37°C with 5% CO_2_. Media remained unchanged for all wells until removal of cells for RNA extraction four days after transfection.

### Cell Proliferation Assay

MCF-7 cells were maintained in 1X RPMI medium supplemented with 10% of FBS and 1% of 1X Streptomycin/Penicillin (Life Technologies, Grand Island, USA). 1×10^4^ cells were plated onto the center of each scaffold (Control, Cdk2i and EGFPi) piece (6.35 mm diameter) (n = 3/scaffold type) that was placed inside a well of a 96 well tissue culture dish. After a 1 hr incubation (allow cells adhere to scaffolds), 100 µl of 1X RPMI medium supplemented with 10% of FBS and 1% of 1X Streptomycin/Penicillin (Life Technologies, Grand Island, USA) were added to each well and the plate was incubated at 37°C with 5% CO_2_. After 24 hr, the cell containing scaffolds were removed and placed in a new 96 well plate and cell proliferation was measure using the MTS reagent (Promega). 20 µl of MTS reagent was added to each well containing 100 µl media. The plates were then incubated at 37°C for 1.5 hr. The scaffolds were then removed and the optical density of the MTS/media solution was determined in an ELISA plate reader (Biotek, EL800) at 492 nm. Values from control wells (MTS/media solution that did not contain scaffolds or cells) were subtracted from each experimental value.

### LIVE/DEAD Assay

Four pieces of 1.0×1.0 cm Control, Cdk2i and EGFPi scaffolds were tested. 3.6×10^5^ cells were placed onto the center of each scaffold piece. The scaffolds were placed inside a 24 well dish and the cells were incubated for 1 hr before 500 µL of 1X RPMI medium supplemented with 10% of FBS and 1% of 1X Streptomycin/Penicillin mix were added. The scaffold pieces were then further cultured for four days at 37°C and 5% CO_2_. Following incubation, the cells were stained with the live/dead assay (LIVE/DEAD® Viability/Cytotoxicity Kit, Invitrogen) following manufacturer’s instructions and imaged with a Zeiss Axiovert 200 microscope equipped with a CCD camera (Zeiss).

### RNA Extraction and Quantitative Real Time PCR (Q-PCR)

MCF-7 cells were seeded either in 24 well dishes or on four 1.0 cm x 1.0 cm pieces of each of the three different scaffolds (Control, Cdk2i and EGFPi) at a density of 5×10^4^ cells/well. After 4 days of culture, RNA was isolated from either cells plated in the tissue culture dish or those on scaffolds using RNeasy kit per manufacturer instructions (Qiagen). The final RNA was treated with DNase (Qiagen) and quantified using a ND-1000 Spectrophotometer (Nano Drop). Q-PCR was carried out using a QuantiTect SYBR Green RT-PCR Kit (One step) (Qiagen) on a LightCycler system (Roche) as previously described by our laboratory [28], [29]. Primers were designed using the template sequence from the human Cdk2 mRNA sequence (Accession# NM_001798) and GAPDH (Accession# M33197). Cdk2 forward primer: 5′ GCC TAA TCT CAC CCT CTC C 3′; Cdk2 reverse primer: 5′CCC TTT CAC CCC TGT ATT CC 3′. GAPDH forward primer: 5′ CAG TCA GCC GCA TCT TCT TT 3′; GAPDH reverse primer: 5′ GCC CAA TAC GAC CAA ATC C 3′. PCR conditions were the following: Reverse transcription at 50°C for 20 min. Amplification: denaturation for 15 sec at 94°C, annealing for 30 sec at 58°C and extension for 30 sec at 72°C, for 40 cycles times. Cdk2 expression was normalized to its corresponding GAPDH value. All Q-PCR products were checked via agarose gel electrophoresis to assess amplification. Each run was replicated at least three times and results were reported as expression level ± sd.

### Statistics

All quantitative data are reported as the average results of three or more independent experiments with sd indicated by error bars. Significance was determined in all analyses by one way ANOVA followed by a Tukey post hoc test.

## Results

### Scaffold Morphology

**Figure 1:**
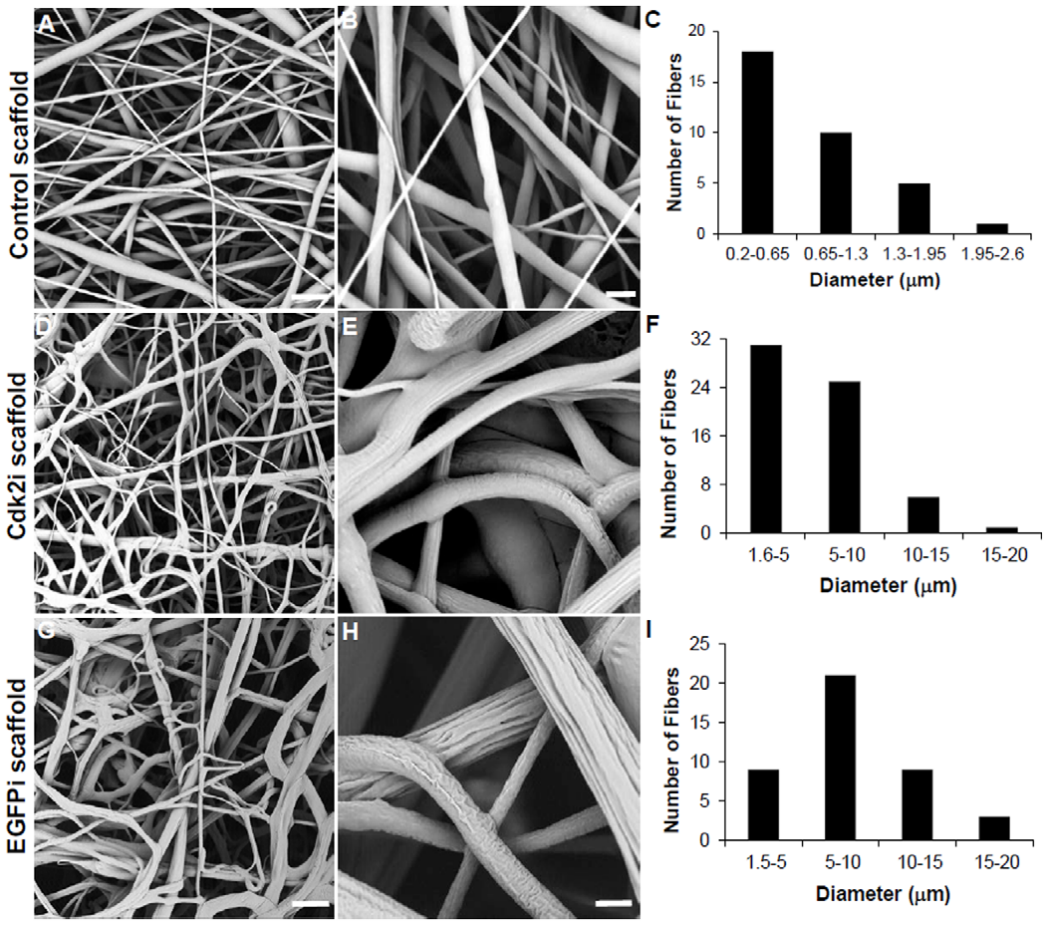
Morphology and fiber diameter of electrospun scaffolds. SEM images of Control scaffolds without DNA (A, B) and those containing plasmid DNA encoding for Cdk2 (Cdk2i) or EGFP (EGFPi) shRNA (D, E and G, H, respectively). C, F, and I indicate the fiber diameter distribution between the three types of scaffolds (using corresponding images in A, C and E). Scale bar in A = 10 µm, B = 2 µm and in D and G = 35 µm, E and H = 5 µm.

All of the scaffolds were electrospun using PCL as the base polymer. The three different types of electrospun scaffolds were: 1) Control (containing no DNA); 2) Cdk2i (containing pKD-Cdk2-v5 plasmid DNA); and 3) EGFPi (containing pKD-EGFP-v1 plasmid DNA, also served as a control). In the Control, the fibers appeared to be smooth, linear and stacked one layer on top of the other, as expected from a nonwoven electrospun scaffold (Fig. 1A and B), with a fiber diameter ranging from 0.2 to 2.6 µm (Fig. 1C). In the plasmid DNA containing scaffolds, the fibers were not linear as the Control and had curves and sometimes tangled fibers (Fig. 1D and G); the surfaces of these fibers were also more irregular and showed grooves (Fig. 1E and H). In addition, the fiber diameter range in the Cdk2i scaffold ranged from 1.6 to 20 µm (Fig. 1F) and in the EGFPi scaffold, from 1.5 to 20 µm (Fig. 1I), which was a magnitude larger than that of the Control scaffold (Fig. 1C). The majority of fibers in the Control scaffold were also submicron in diameter (Fig. 1B), whereas with both DNA containing scaffolds they were up to 10 µm (Fig. 1F and I).

### DNA Release

**Figure 2:**
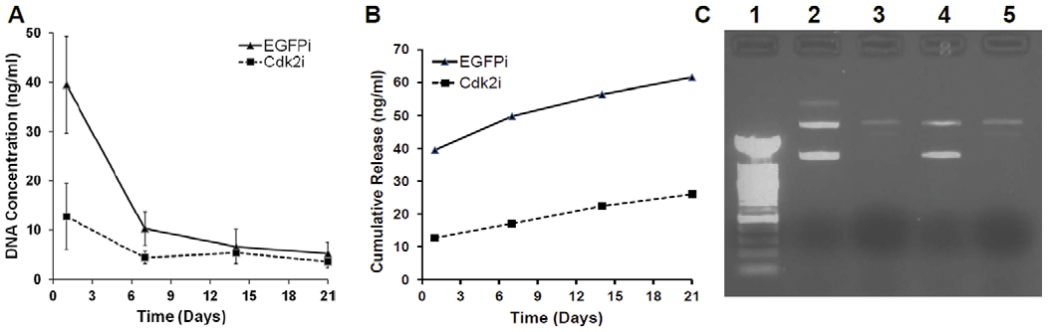
Plasmid DNA release from electrospun scaffolds. Graphs indicates the amount of DNA release per time point (A) and cumulative release (B) over the 21 day study from the two plasmid DNA containing scaffolds, Cdk2i and EGFPi. (C) Agarose gel electrophoresis showing the integrity of the released DNA from both scaffolds. Lane 1, 100 bp MW marker; Lane 2 and 4, EGFP (pKD-EGFP-v1) and Cdk2 (pKD-Cdk2-v5) control (unincorporated) plasmid DNA; Lane 3 and 5, 21 day released EGPF and Cdk2 plasmid DNA, respectively.

For both EGFPi and Cdk2i scaffolds, the concentration of DNA released within the first 24 hours was much greater than the amount released over the following three weeks (Fig. 2A), representing a burst of DNA release that was observed with our previous studies using plasmid DNA containing electrospun scaffolds [12], [13]. The amount of plasmid DNA released from the EGFPi scaffold was 2–3X greater than the amount released from the Cdk2i scaffold (despite the plasmid DNA being the same size; 3,099 bp) at the first two time point measured (Fig. 2A), as well as cumulatively (Fig 2B). Further, the integrity of the released plasmids (retrieved from the 21 day experiment) was determined by gel electrophoresis (as compared to the unincorporated plasmid DNA [500 ng/lane], Fig. 2C, Lanes 2 and 4– top prominent band indicates linear plasmid while the bottom shows supercoiled plasmid DNA); results showed that the integrity of the released EGFP and Cdk2 plasmid DNA was maintained through the electrospinning process (Fig. 2C, Lanes 3 and 5, respectively). But, while the top band (linear plasmid DNA) for each of the released plasmids (Fig. 2C, Lanes 3 and 5) was the same as that of the control EGFP and Cdk2 plasmid samples (Fig. 2C, Lanes 2 and 4, respectively), the bottom band (supercoiled plasmid) was absent in the released DNA samples (Fig. 2C, Lanes 3 and 5). Also, the weak intensity of the released DNA bands (Fig. 2C, Lanes 3 and 5) reflected the low amount of total DNA released from each of the scaffolds at the 21 day time point and was consistent with the quantitative measurements (Fig. 2B). Lastly, the total amount of DNA released was calculated to be ∼2.5%.

### Scaffold Degradation

**Figure 3:**
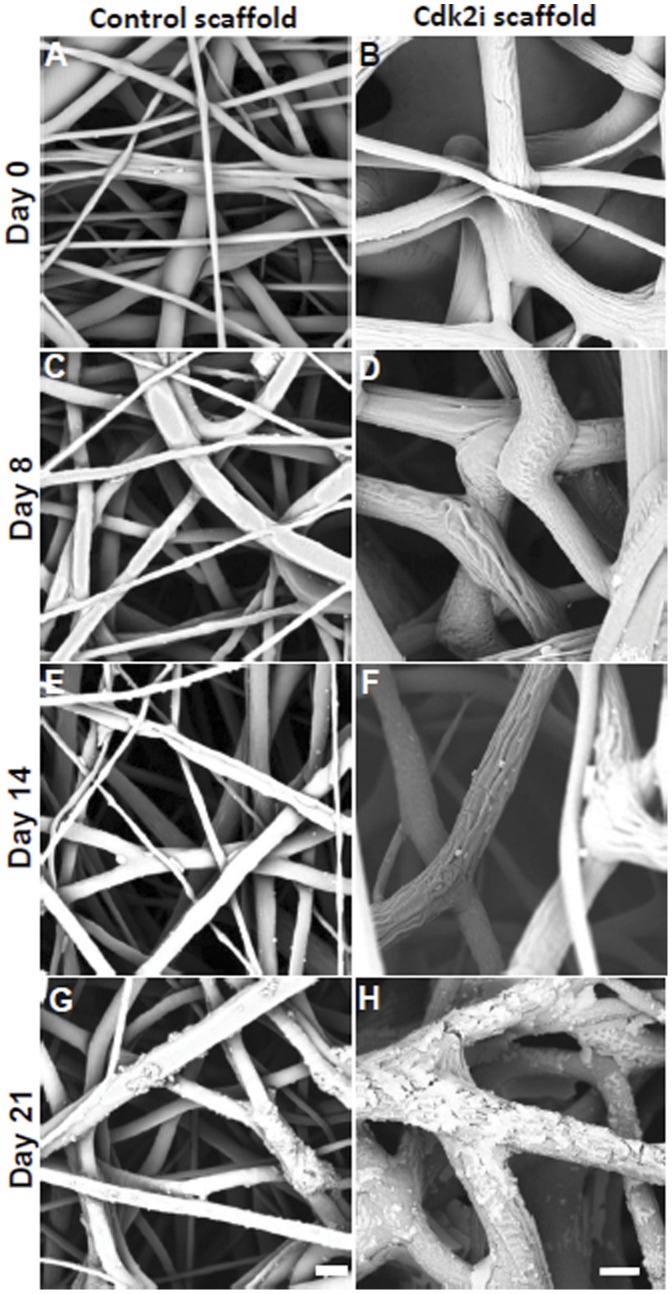
Electrospun scaffold degradation. SEM images of Control scaffolds without DNA (A,C,E,G) and with scaffold containing plasmid DNA encoding for Cdk2 shRNA (Cdk2i, B, D, F, H) at the specified days (0, 8, 14 and 21). Scale bar in A, C, E, G = 2 µm, and in B, D, F, H = 5 µm.

Since the amount of the plasmid released from the scaffolds over a three week period was very low, the degradation of the fibers from the Cdk2i electrospun scaffold, as well as the Control, was examined for the same time. After 8 (Fig. 3C and D) and 14 days (Fig. 3E and F), the DNA loaded scaffold, Cdk2i, and the Control showed no sign of degradation (the groves that appear on the fibers from the Cdk2i scaffold at day 8 and 14, Fig. 3D and F, respectively, are normal and do not reflect degradation). The fiber networks were still intact and the morphology of individual fibers remained unchanged as those at the start of the experiment (day 0, Fig. 3A and B). However, after 21 days, both scaffolds showed signs of fiber degradation (Fig. 3G and H). Specifically, while the degradation of the Control scaffold fibers was evident (Fig. 3G), those of the Cdk2i scaffold displayed major cracks and severe surface “scales” or “flakes” (Fig. 3H), especially as compared to those shown at day 0 (Fig. 3A and B).

### Silencing of Cdk2 via Plasmid DNA Encoding for shCdk2 RNA

**Figure 4:**
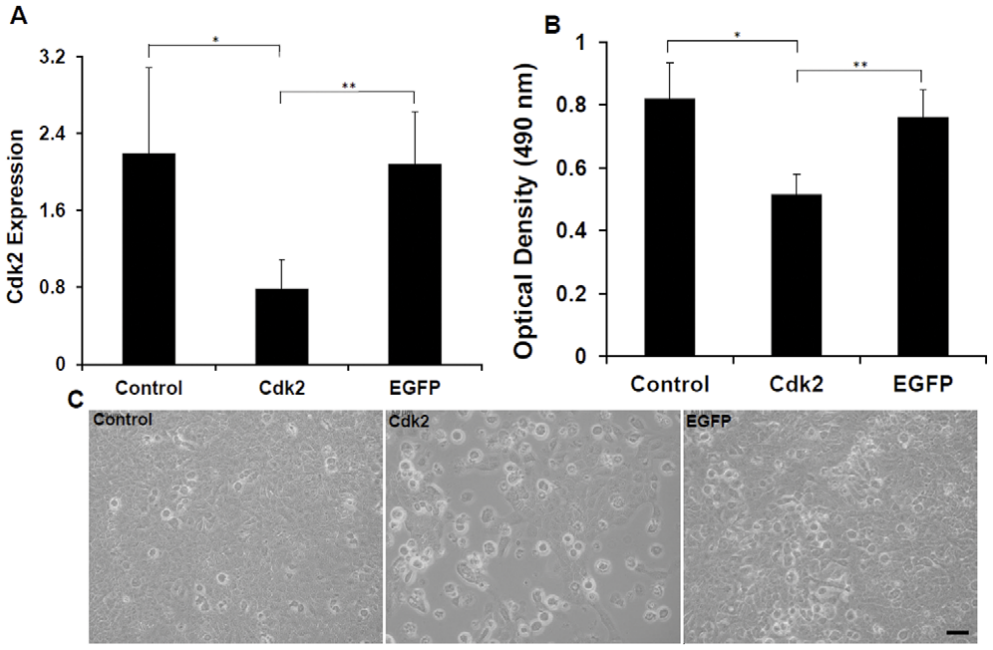
Silencing of Cdk2 and suppression of MCF-7 cell proliferation. The effectiveness of the plasmid DNA encoding Cdk2 shRNA was tested on MCF-7 cells growing on tissue culture plates, both in terms of suppressing Cdk2 expression, as measured by Q-PCR on culture day 4 (A) and cellular proliferation, as measured by the MTS assay on culture day 1 (B). The effect on proliferation and cell death was also examined via microscopy in the three conditions (C). The plasmid DNA encoding for EGFP shRNA was used as a control in both experiments. Control indicates results from untransfected cells. (A) *p<.03, **p<.05. (B) *p<.001, **p<.001.

To establish a baseline for comparison to the scaffolds, the effectiveness of the Cdk2 shRNA encoding plasmid (pKD-Cdk2-v5) was checked by transfecting MCF-7 breast cancer cells growing in culture. As a control we also used untransfected cells or those transfected with the control EGFP shRNA encoding plasmid (pKD-EGFP-v1). We performed two analyses to verify how effective the encoded shCdk2 RNA was in silencing Cdk2; measuring Cdk2 mRNA via Q-PCR and cell proliferation. Q-PCR of Cdk2 mRNA expression was conducted on RNA isolated from the untransfected cells and those transfected with the DNA plasmids to determine the efficiency of the Cdk2 shRNA in silencing its target gene. The EGFP plasmid was used as a control. Results from these experiments showed that the level of Cdk2 mRNA expression was significantly lower,∼65% and 63%, in the cells transfected with the pKD-Cdk2-v5 plasmid than in the control with untransfected cells (p<0.03) or those transfected with the pKD-EGFP-v1 plasmid (p<0.05), respectively (Fig. 4A). These results mirrored those with the proliferation assay; cell proliferation with the Cdk2 shRNA encoding plasmid was significantly lower, ∼38% and 33% than that of both, the control (p<0.001) and the EGFP shRNA encoding plasmid (p<0.001), respectively (Fig. 4B). Cell proliferation was also monitored qualitatively using microscopy and showed the same decrease in cell number in the presence of the Cdk2 shRNA encoding plasmid (Fig. 4C, middle panel), in contrast to those of either control untransfected cells (Fig. 4C, left panel) or transfected with the EGFP shRNA encoding plasmid (Fig. 4C, right panel). In addition, there were many round cells present with the Cdk2 shRNA encoding plasmid, indicating the presence of dead cells (Fig. 4C, middle panel). In contrast, there were hardly any round cells in the other two conditions, control and with the EGFP plasmid (Fig. 4C, left and right panels). These analyses serve as a baseline for comparing the effects of the plasmid DNA scaffolds in silencing Cdk2 mRNA and suppressing cell proliferation.

### Silencing of Cdk2 via Scaffold Containing Plasmid DNA Leading to Suppression of Cell Proliferation and Increase Cell Death

**Figure 5:**
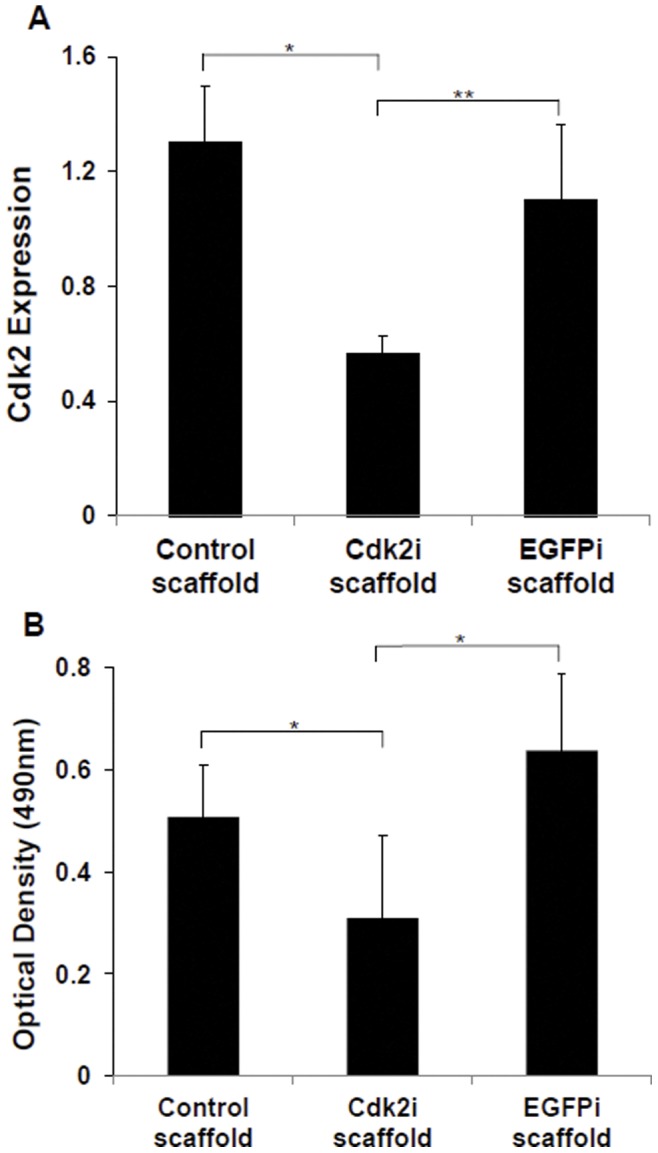
Silencing of Cdk2 and suppression of cell proliferation by the Cdk2i scaffold. The effectiveness of the plasmid DNA containing scaffolds was tested on MCF-7 cells growing directly on them. Control scaffold and those containing plasmid DNA encoding for Cdk2 (Cdk2i) or EGFP (EGFPi) shRNA, both in terms of suppressing Cdk2 mRNA, as measured by Q-PCR on culture day 4 (A) and cellular proliferation, as measured by the MTS assay on culture day 1 (B). The Control and EGFPi scaffolds served as controls in both experiments. (A) *p<.01, **p<.04. (B) *p<.02, **p<.001.

Given the above results that established the baseline effectiveness of the plasmids in silencing Cdk2 and suppressing cell proliferation and viability, we next examined whether our shRNA plasmid DNA containing scaffolds were able to perform the same way by plating cells directly on the three different types of scaffolds (Control, EGFPi, and Cdk2i). The level of Cdk2 mRNA expression was again significantly lower (∼57% and 50%) on cells plated on the Cdk2i scaffold than those on the Control (p<0.01) or EGFPi scaffold (p<0.04), respectively (Fig. 5A). There was no significant difference in Cdk2 expression between the Control and EGFPi scaffold (Fig. 5A). Similarly, there was a significant decrease (∼40% and ∼52%) in cell proliferation on the Cdk2i scaffold in comparison with that of both the Control (p<0.02) and EGFPi (p<0.001), respectively (Fig 5B). And again, there was no significant difference in Cdk2 expression between the Control and EGFPi scaffold (Fig. 5B). Collectively, the Cdk2 expression and proliferation data obtained with the scaffolds (Fig. 5) mirrors that obtained with cells grown on tissue culture wells (baseline conditions, Fig. 4).

**Figure 6:**
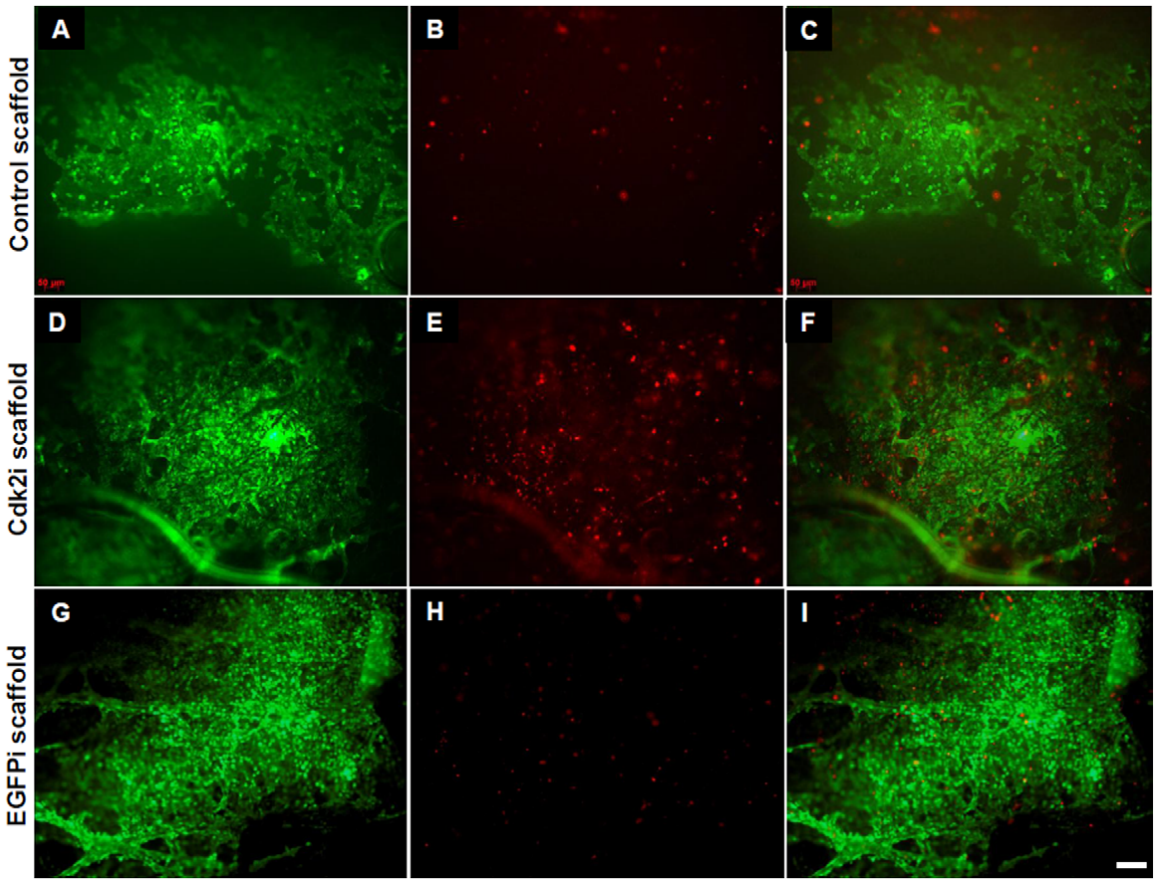
Silencing of Cdk2 and cell death by the Cdk2i scaffold. LIVE/DEAD assay of MCF-7 cells grown on each of the three scaffolds, Control (A, B, C), and those containing plasmid DNA encoding for Cdk2 (Cdk2i, D, E, F) or EGFP (EGFPi, G, H, I) shRNA. A, D, H show living cells (green); B, E, H show dead cells (red); C, F, I represent the overlay of the two corresponding red/green images. Scale bar = 50 µm.

To investigate whether the observed decrease in cell proliferation was due to the disruption of the cell cycle and even leading to cell death, we utilized the LIVE/DEAD assay. Qualitative analyses of the cells plated on the three types of electrospun scaffolds clearly show that there was an increase in the number of dead cells on the surface of the Cdk2i scaffold (Fig. 6E) than those plated on the Control (Fig. 6B) or EGFPi scaffold (Fig. 6H). In addition, a considerable amount of live cells were present in all three scaffolds (Fig.6A, D, G). Lastly, the qualitative ratio between the live and dead cells can easily be seen in the overlay panels for all three scaffolds (Fig. 6C, F and I).

## Discussion

The present study successfully demonstrated the effective silencing of Cdk2 mRNA and subsequent suppression of breast cancer cell proliferation with an increase in cell death via the use of an electrospun scaffold containing plasmid DNA encoding for shRNA targeting Cdk2. To our knowledge, this is the first demonstration of such an experimental approach to disrupt cancer cell growth using electrospun plasmid DNA containing scaffolds. Other studies have also explored the use of nonviral delivery of siRNA, microRNAs, and shRNAs through polycation, liposomal, and biochemical chaperoning [5]. Specifically, these studies used a variety of platforms to deliver the siRNA or shRNA and ranged from hydrogels [30]–[34] films [35], [36], nanoparticles [37]–[39], sponges [40]–[41], and even creams [42]. Collectively, these studies targeted various genes that play a role in a variety of biological processes.

More relevant to this work, only two studies (from the same group) were previously published and utilized electrospinning to create fiber based scaffolds for delivering, not plasmid DNA, but siRNA oligonucleotides [26], [27]. The initial study utilized PCL alone or PEGylated as the base polymer while the second study also included a copolymer of caprolactone and ethyl ethylene phosphate (PCLEEP); both studies incorporated siRNA oligunucleotides to functionalize the electrospun nanofibers which displayed fiber diameters that ranged between 300–400 nm. Similarly, our control scaffold with only PCL and no plasmid DNA, revealed that the majority of fibers had diameters between 200–600 nm. In contrast, our electrospun scaffold containing PCL and plasmid DNA exhibited fiber diameters that ranged between 1–20 µm (with the majority in the range of 1–10 µm), signifying the applicability of a relatively broader range fiber diameters and with the presence of grooves. The grooves most likely resulted from the fact that PCL and DNA are not truly miscible polymers.

Previous studies in our laboratory using plasmid DNA and PLGA with a PLA-PEG block copolymer resulted in submicron sized fibers, but when the concentration of the block copolymer was increased from 10 to 15%, we also observed a much thicker (∼2–5 µm) fiber diameter [12]. The changes in the fiber diameter from mixed polymers in solution during the electrospinning process are often due to variation in the polymer characteristics and the rate of evaporation of the solvent mixture. In the current context, the fiber diameter has little or no significant effects to the conclusions of the study, but clearly could affect the release kinetics of the plasmid DNA at longer periods of time and consequently the duration of silencing.

The DNA release profile of the Cdk2i scaffold under physiological conditions (in saline buffer at 37°C with agitation) showed a burst of release at the initial 24 hrs which declined with time, and is in agreement with our previous results [12], [13]. Even though, we observed the beginning of fiber degradation around 21 days in physiological conditions, no additional burst release of DNA was observed. Surprisingly, and although the same amount of DNA was incorporated into the scaffolds, ∼2–3X more DNA was released from the EGFPi scaffold as compared to the Cdk2i. Since the size of the plasmids, electrospinning parameters, base polymer and starting amount of DNA were all the same, this discrepancy in the total amount of released plasmid DNA between the two different types of scaffolds remains to be elucidated. Additionally, the total amount of plasmid DNA released was low, (∼2.5%), consistent with the previous study of Cao and colleagues [26], who reported a DNA release of ∼3% using electrospun PCL alone. Clearly, the bulk of the plasmid DNA was trapped within the fibers and very little was on the surface, which probably accounted for the low amount released. However, the release profile and amounts of plasmid DNA can be modified and increased, respectively, through the inclusion of a block copolymer as previously demonstrated in our laboratory with DNA release rates of ∼80% [12]. Similarly, when Rujitanaroj and colleagues [27] included a copolymer of caprolactone and ethyl ethylene phosphate (PCLEEP) to fabricate their PCL based scaffolds, the siRNA oligonucleotide release was increased to ∼89–97% (based on a 49 day study). Regardless, the more important point is that the plasmid DNA was released from both of our scaffolds and was structurally intact and bioactive as demonstrated by the gel electrophoretic analysis and functional cell based assays, again consistent with our previous studies [12], [13], as well as those of others using DNA containing electrospun scaffolds [17], [21], [24].

Unlike the previous aforementioned studies [26], [27] that targeted a housekeeping gene (i.e. GAPDH) in combination with a transfection reagent to demonstrate the effectiveness and efficacy of their nanofibrous electrospun scaffold-mediated siRNA, we specifically wanted to go beyond the “proof of concept” aspect of an electrospun scaffold as a RNAi delivery system and demonstrate a real biological application. As such, we designed our electrospun scaffolds to deliver plasmid DNA encoding for shRNA targeting Cdk2, a cell cycle specific protein, coupled to a cellular based assay, i.e. proliferation and cell death, using the human breast cancer cell line, MCF-7. Further, Cao et al [26] showed that plating cells directly on the PCL siRNA based scaffolds resulted in a 22.3% silencing efficiency for GAPDH mRNA (despite a 3% siRNA release rate). Similarly, in our case, even with only a ∼2.5% plasmid DNA release from the scaffolds, the suppression level of Cdk2 mRNA (as measured by Q-PCR) by the Cdk2i scaffold was ∼57% and was comparable to that of directly transfecting cells with the shRNA Cdk2 unincorporated control plasmid which showed a ∼65% silencing efficiency (baseline levels). Clearly then, small amounts of released plasmid DNA encoding for shRNA are sufficient to suppress their target gene at over 50% of mRNA levels. More importantly, this level of target gene suppression was also translated into a real biological effect, i.e. decrease cellular proliferation and increase cell death. Whereas in the Cao study, cellular proliferation was found to be unchanged when HEK 293 cells were plated directly on PCL scaffolds containing siRNA oligonucleotides [26], on our Cdk2i PCL based scaffold, the proliferation rate of MCF-7 cells decreased by ∼40% and 52% in comparison to cells plated on the Control or EGFPi scaffolds, respectively, within 24 hrs. More importantly, this 40% suppression level of cell proliferation was also comparable with that of directly transfecting cells with the Cdk2 shRNA encoding plasmid which resulted in a ∼37% decrease (baseline level). Lastly, and consistent with our quantitative measurements of cell proliferation, using the LIVE/DEAD assay, we did observe a higher number of dead cells on the Cdk2i scaffold in comparison with those on the Control and EGFPi scaffold, leading us to conclude that the shRNA targeting Cdk2, not only suppresses cell growth by arresting the cell cycle, consistent with previous studies [43], but it also led to cell death. But the exact mechanism of cell death (i.e. apoptosis, authophagy, necrosis) remains to be determined.

This is clearly a robust decrease in cell proliferation and increased cell death given the small amount (∼2.5%) of plasmid DNA released by the scaffolds. Undoubtedly, if we increase the amount of released plasmid DNA, we would expect to have a greater silencing efficiency on Cdk2 and thus a stronger suppression of cellular proliferation and cell death. In fact, Rujitanaroj and colleagues^27^, demonstrated exactly this by incorporating a copolymer of caprolactone and ethyl ethylene phosphate (PCLEEP) as well as a transfection agent into their PCL scaffolds containing siRNA oligonucleotides targeting GAPDH and showed both, an increase in silencing efficiency, as well as a decrease in cell viability.

Taken together, these results clearly demonstrate the effectiveness of the use of electrospun scaffolds as DNA delivery systems. Further, our study validated the approach of utilizing a plasmid DNA (encoding for a specific shRNA) to silence a target gene (i.e. Cdk2), disrupt the cell cycle and negatively regulate the proliferation of cancer cells (MCF-7), ultimately, leading to cell death. Certainly, future modifications in this approach will alter the design of the scaffold in order to “personalize” the controlled and sustained delivery of si or shRNA to various tumors and effectively destroy the cancer cells. The next step is to be able to test the *in vivo* efficacy of plasmid DNA or oligonucleotide based electrospun scaffolds in their ability to deliver enough si or shRNA in order to successfully silence their target genes and destroy the tumor cells. As such, the true potential of scaffold mediated delivery of RNAi is yet to be fully realized and thus, additional research is strongly warranted.

### Conclusions

Results from this work indicate that it is possible to fabricate an electrospun scaffold with the ability to successfully release plasmid DNA encoding for shRNA, with high silencing efficiency and subsequent suppression of cancer cell proliferation and increased cell death. To our knowledge, this is the first demonstration of utilizing a plasmid DNA based approach coupled to polymeric electrospun scaffolds for RNAi. The plasmid DNA is easily incorporated and released from the scaffold, but more importantly, the DNA remains bioactive and capable of cell transfection, transcription and translation, and ultimately silencing its target gene. This new application of electrospun scaffolds could serve as a novel biomaterial approach and a new opportunity in the non-viral delivery of si, sh or microRNAs targeting various genes and specific biological processes involved in cancer treatment, as well as tissue engineering and regenerative medicine.
